# A Study on the spatial form of traditional villages in jiangnan region of china from the perspective of human thermal comfort: A case study of nanjing, jiangsu province

**DOI:** 10.1371/journal.pone.0323252

**Published:** 2025-05-09

**Authors:** Yao Xiong, Yinhui He, Xinyu Xie, Tingting Zhai, Ningling Chu, Lili Shen, Yunfeng Yang

**Affiliations:** College of Art & Design, Nanjing Forestry University, Nanjing, China.; Yunnan University, CHINA

## Abstract

Traditional Chinese villages embody the ecological wisdom of ancient people to “conform to nature and transform nature”. In the long-term process of natural evolution, the natural environment and human production and living space have been combined to develop a unique spatial pattern with climate adaptability, which has the ability to cope with and regulate natural climate. Under the context of China’s rural revitalization, a study into the microclimate and thermal comfort of traditional villages holds promise for fostering the development of ecologically sustainable and aesthetically pleasing rural communities. This study focuses on three representative traditional villages in Nanjing. By employing a combination of measured data and ENVI-met numerical simulation, the microclimate effects of distinct spatial domains in these villages are analyzed. Additionally, the thermal comfort PET values are calculated using the Rayman platform, thereby objectively examining the relationship between spatial configuration and microclimate factors in Jiangnan traditional villages. The findings reveal that the PET values range from 38.4 to 57°C in summer and from 0.1 to 27°C in winter, with winter thermal comfort generally surpassing that of summer. From various villages. the internal water system of Shishanxia Village is scattered and the space is dense, with good ventilation and balanced humidity. Therefore, its thermal comfort in winter and summer is optimal. The northern mountain of Huanglongxian Village can effectively block the northwest monsoon and form a wind barrier, which can achieve the effect of keeping warm and controlling temperature in winter. Therefore, Huanglongxian has better thermal comfort in winter. Huashu Village is surrounded by water systems, with dense internal buildings and large hard areas inside the village. Plants are scarce, which can easily cause local high temperatures due to the absorption and radiation of solar radiation by hard underlying surfaces and buildings. Therefore, the comfort in winter and summer is the worst. Finally, the spatial configuration and landscape elements that influence human thermal comfort are revealed and transformation strategies tailored to each space type are summarized, aiming to provide scientifically grounded and rational recommendations for climate-adaptive design in rural areas.

## 1. Introduction

Approximately half of the global populace resides in rural regions, with China having the highest number of villages worldwide. According to the findings of the seventh national census conducted in 2021, China harbors a staggering 69,000 administrative villages, 2.617 million natural villages, and a rural population of 500 million, constituting 36.11% of the overall populace. Since the advent of the 21st century, the Chinese government has promulgated 21 central documents centered around the pivotal theme of “*agriculture, rural areas and farmers*,” thereby formulating precise plans and arrangements for the construction and advancement of rural areas. Concurrently, with the rapid progression of urbanization in China, the traditional way of life has gradually transitioned towards modernity, the environmental predicaments that plague urban areas have increasingly appeared in rural regions, and the overexploitation of natural resources have ravaged the original architectural fabric and aesthetic appeal of rural areas, resulting in severe harm to the rural ecological environment and exacerbating the burden of global warming [1].

In recent years, under the auspices of the rural revitalization strategy, the Chinese government has underscored the significance of “building ecologically livable beautiful villages, continuously improving rural public infrastructure and improving rural human settlements environment are important tasks for implementing the rural revitalization strategy.” The challenges faced by rural areas, such as inadequate infrastructure and unsanitary surroundings, have been duly addressed. Nevertheless, China’s ongoing fervent pursuit of new rural construction primarily focuses on spatial layout, cultural aspects, and infrastructure, while neglecting climate adaptability and the specific methodologies required to achieve it. Therefore, it is necessary to steer contemporary village construction practices by leveraging pertinent research results. Thus, enhancing the microclimate environment of villages through the implementation of effective environmental design strategies and the reasonable utilization of existing natural resources and climatic conditions emerges as a crucial prerequisite for attaining the green development of rural areas. Moreover, it represents a pivotal task and subject matter in the current rural revitalization strategy.

Thermal comfort denotes the state of satisfaction experienced by individuals in a localized climate environment, as measured through human thermal comfort [2]. The definition of human thermal comfort is “the subjective satisfaction evaluation that humans make on their surrounding thermal environment.” [3] This definition serves as a direct reflection of human perception of the environment and remains a focal point in current research on outdoor microclimates [4,5]. In recent years, the exacerbation of the heat island effect has prompted the field of human settlement environment science, with architecture, urban planning, and landscape architecture at its core, to pay special attention to design strategies and technologies based on climate adaptability. In 2011, AHA Mahmoud conducted a measurement and evaluation of microclimate parameters and thermal comfort in Cairo Park’s landscape space, highlighting the significant impact of sky view factor and wind speed on thermal comfort evaluation [6]. In 2014, D Govindarajulu studied the regulatory effect of urban green spaces on urban microclimate in India, emphasizing the economic and effective measure of prioritizing urban green spaces to adapt to climate change [7]. In 2017, A. Santos Nouri analyzed the microclimate factors affecting the pedestrian thermal comfort threshold in square spaces using numerical model simulation methods, further exploring the transformation of these factors into design elements for creating thermally comfortable square space [8]. In 2020, Martina Petralli evaluated human thermal comfort in Cascine Park in Florence using physiological equivalent temperature and identified the significant impact of shading areas on the thermal comfort value of the site space [9]. Most research on microclimate and thermal comfort primarily focuses on urban space studies [10–12], specifically studying different urban public spaces such as parks [13], squares [14], streets [15–17].

In recent years, the proposal of China’s rural revitalization strategy has garnered increasing attention towards the preservation and rejuvenation of villages [18,19]. Scholarly focus has primarily centered around the design of residential buildings [20], spatial distribution in settlements [21], and the development of village tourism [22–24]. Notably, in 2012, Zhang Qian explored the correlation between traditional settlement forms and climate in southeastern Hubei based on data measurement and type induction methods[25]. In 2017, Lu et al. conducted measurements and quantitative analyses to consolidate the construction wisdom and adaptability strategies observed in traditional villages and residences in South China [26]. Subsequently, in 2018, Qi’s research team explored the influence of the mountain-water pattern on the microclimate environment in typical ancient villages situated in the Beijing-Tianjin-Hebei region, employing measurements and numerical simulations to substantiate their findings. Their studies demonstrated that a mountain-water pattern, encompassed by surrounding mountains, effectively obstructs the ingress of adverse climatic factors into villages [27]. Similarly, in 2019, Liu employed GIS, Rhino-grasshopper, and other software to appraise 36 traditional settlement samples in Zhejiang Province. This study sought to explore the correlation between rural public spaces and the microclimate environment, finally summarizing the green construction experiences derived from traditional settlements [28]. In addition, in 2020, Qi conducted a comparative study on traditional Chinese villages, aiming to explore the ecological wisdom inherent in the design of ecologically adaptable traditional villages, while also summarizing strategies for the development of ecologically-oriented village tourism [29]. In the same year, Pang Xinyi used qualitative and quantitative methods, as well as interdisciplinary research, to study the forms of villages, streets, and courtyards. Finally, she proposed climate adaptive optimization design strategies for village spaces[30]. In 2022, Guo utilized PHOENICS, based on the extension correlation function, to evaluate the climate comfort of traditional village squares, subsequently proposing optimization design strategies [31]. Similarly, in the same year, Zhang employed ArcGIS to classify 1194 Linpan settlements in Chengdu. By utilizing ENVI-met to simulate the statistical results and employing orthogonal experiments to obtain 25 distinct Linpan spatial forms, they explored the most comfortable Linpan layout [32]. Presently, research on the microclimate of villages in China predominantly adopts a macro perspective, assessing the relationship between site selection and the landscape pattern of regional villages [25,27]. Conversely, other studies adopt a micro perspective, focusing on the spatial or residential architectural morphology of individual villages [28,33,34]. However, these single case studies fail to provide universal conclusions, as they lack cross-analysis and comparative analysis of multiple cases of the same type, thereby limiting the depth of research.

For the aforementioned analysis, this study will utilize Nanjing as a case study, wherein three exemplary traditional villages will be selected. Through the utilization of field measurements and numerical simulations, a quantitative study of the microclimate factors within rural spaces will be conducted from both a meso and micro perspective. The objective thermal comfort of each space will be assessed by means of RayMan, which calculates the physiological equivalent temperature (PET). In addition, the thermal comfort of distinct space typologies will be explored through ENVI-met simulation analysis. The primary objective of this research is to appraise the climate adaptability of the internal spaces in traditional villages, thereby addressing the existing research gap pertaining to rural climate thermal comfort. The findings of this study have the potential to offer novel insights for enhancing the living environment and facilitating the design of climate-adaptive measures for traditional villages across diverse regions.

## 2. Materials and methods

Traditional villages have undergone a long process of evolution, wherein they have integrated natural circumstances with the exigencies of production and habitation, thereby constantly coordinating the relationship between individuals, villages, and the environment. Therefore, traditional villages have developed a spatial configuration endowed with unparalleled climatic adaptability, encapsulating the ecological wisdom of ancient Chinese progenitors who “adapt to nature and transform nature,” while simultaneously embodying the symbiotic ethos of “harmony between man and nature” in traditional Chinese Feng Shui doctrine. The spatial arrangement of traditional villages in the southern region of the Yangtze River is profoundly influenced by the principles of traditional Feng Shui theory. From the external environment of the natural surroundings to the internal spatial structure of the village, and even extending to the construction of residential buildings, all facets bear indelible imprints of the traditional Feng Shui theory. Notably, in the selection of village sites, attention is paid to the landscape arrangement characterized by being “surrounded by mountains and water, facing water with mountains behind, negativing yin and embracing yang, hiding wind and gathering qi.” This holds immense value for the research of design principles pertaining to rural climate adaptability.

This paper delves into the correlation between distinct spatial form characteristics and the microclimate environment in three provincial-level traditional villages situated in the southern region of the Yangtze River, namely Huanglongxian Village, Shishanxia Village, and Huashu Village. Through the utilization of field measurements and numerical simulations, an exploration is conducted to identify the impact of spatial form and landscape elements on human thermal comfort. The findings of this research seek to offer novel insights for the design of rural spaces in diverse geographical contexts.

### 2.1 Research area

**Fig 1:**
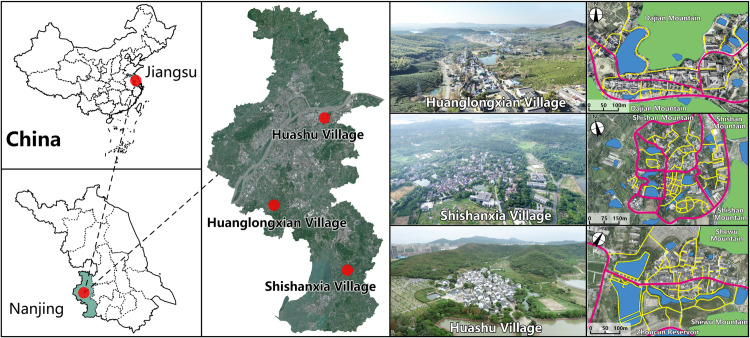
Research area (village photo taken by drone).

This study has chosen three traditional villages situated in Nanjing, Jiangsu Province (Fig 1). Nanjing, located in the southern region of the Yangtze River in China, is characterized by its hilly terrain, predominantly consisting of low mountains and gentle hills. The city is traversed by the Ningzhen Mountains and Laoshan Mountains, which intersect its central area. The southern part of Nanjing is marked by its undulating landscape. The Yangtze River flows through the entire territory, spanning from the southwest to the northeast, while the Qinhuai River and Jinchuan River meander around it. The region has a total of 120 rivers of varying sizes, with the water area accounting for more than 11%, thereby creating a multifaceted and diverse landscape environment. Nanjing experiences a typical North subtropical monsoon humid climate, characterized by a minimum average temperature of -5°C in January, resulting in a winter season lasting 135 d. Conversely, the highest average temperature of 41°C occurs in August, contributing to a summer season lasting 94 d. These climatic conditions exemplify the distinct features of prolonged winters and summers, accompanied by relatively shorter spring and autumn periods.

Huanglongxian Village, Shishanxia Village, and Huashu Village are situated amidst a picturesque landscape of mountains and water, adorned with verdant forests and bamboo groves, seamlessly blending with the natural elements that envelop them. Huanglongxian Village (31°78’76.6”N 118°68’21.3”E) is situated in the Jiangning District of Nanjing, nestled at the base of Dajianshan Mountain. The mountain is located on both sides of the village, north and south, the mountain range runs in an east-west direction, the nearest mountain has an elevation of about 77 meters. the village features a topography that ascends in the north and descends in the south, with a central 2hm^2^ pond gracing its core. Shishanxia Village (31°51’81.4”N 119°06’84.1”E) is located in the southwestern region of Lishui District, westward of Shijiu Lake, southward of Shishan Mountain, and eastward of the mountain range, approximately 400 m from its foothills. The mountain range runs north-south, with an altitude of about 50m. The village exhibits an elevation that rises in the east and lowers in the west, with an assortment of pond patches dispersed throughout its expanse, there are more water bodies on the south side. Huashu Village (32°11’31.2”N 119°03’49.5”E) finds its abode at the western foothills of the Baohua Mountain Scenic Area in Nanjing. It is nestled against the Shewu Mountain Range, the mountain range runs in an east-west direction, with the highest peak reaching an elevation of 281 meters and the closest mountain range to the village being 98 meters. Adjacent to the Zhoucun Reservoir in the south, and bordered by a sizable pond in the west, the overall water area is concentrated on the southwest sides of the village.

From the perspective of the external landscape pattern, these three villages all belong to villages nestled by mountains and water, reflecting the landscape style of villages in the Jiangnan region, in line with the feng shui demands of “embracing mountains and water” and “gathering wind and energy”. Huanglongxian Village is located between two mountain valleys, with a significant elevation difference within the village. Shishanxia Village is located at the foot of Shishan Mountain, with a relatively flat terrain. Huashu Village is located at the foot of Shewu Mountain. The village is high in the north and low in the south, with little terrain fluctuation. The water systems in Huanglongxian Village and Huashu Village present a combination of planar, linear, and point like shapes, while Shishanxia Village presents a combination of point like and linear shapes. The water bodies in Huanglongxian Village and Shishanxia Village are dispersed, while the water system in Huashu Village surrounds the south side of the village in a semi enclosed shape. From the perspective of the village’s planar form, Huanglongxian Village has a combined shape, while Shishanxia Village and Huashu Village have a clustered shape. From the perspective of the internal spatial pattern of the village, both Huanglongxian Village and Huashu Village have a main street, with irregular free and branch shaped street structures. The street structure of Shishanxia Village is grid shaped. Overall, this article selected villages of the same type but with different spatial characteristics for research, in order to provide scientific and reasonable suggestions for the climate adaptability design of mountainous and hilly villages in the Jiangnan region.

### 2.2 Field measurement

#### 2.2.1 Measurement instruments and methods.

By referencing historical meteorological data and the actual meteorological conditions of Nanjing in 2022, representative meteorological days were selected during the coldest month (January) and the hottest month (July) of winter and summer, respectively. Monitoring was conducted for 5 d, from January 15–19, 2022, and from July 9 to July 13, 2022, for 10h per day (8:00–17:00). The average value of the 5 d was then used as the hourly data for each measurement point [35–37]. The specific measurement instruments are listed in Table 1. The measurements were conducted at fixed observation points, with the instruments positioned at a height of 1.4m above the ground. Data was recorded every hour. Additionally, the average meteorological data for these 5 d were obtained from the official records of the Nanjing Meteorological Station, serving as external references and initial simulation values.

**Table 1:** Experimental equipment used for the measurement ofmicrometeorological parameters.

Measured Parameter	Instrument	Range	Accuracy
Air temperature	DHM2A	-26°C ~ 51°C	≤ ± 5%
Relative humidity	DHM2A	10% ~ 100%	±3%
wind speed	Fluck 925	0.40 ~ 25.00 m/s	±2%
Globe temperature	AZ8758	0% ~ 50%°C	±3%
spatial distance of the site	LM50	0 ~ 50m	±4%

#### 2.2.2 Measurement point setting.

**Fig 2:**
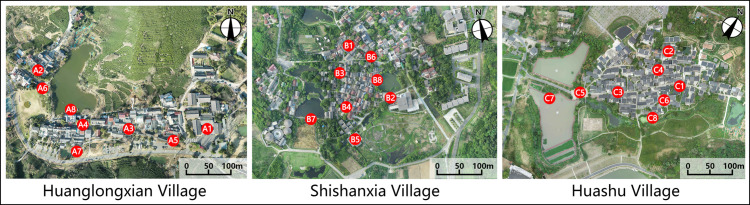
Positioning map of each measurement point in the village.

**Fig 3:**
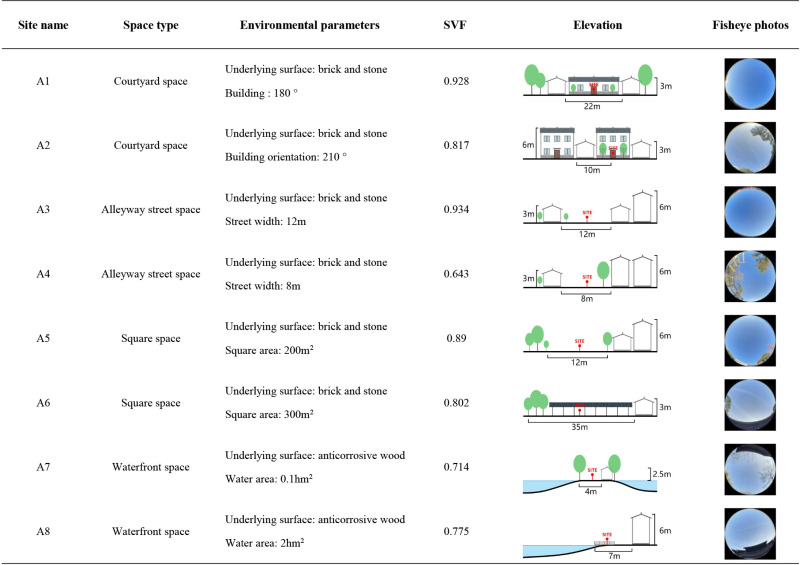
Measurement point settings in Huanglongxian Village.

**Fig 4:**
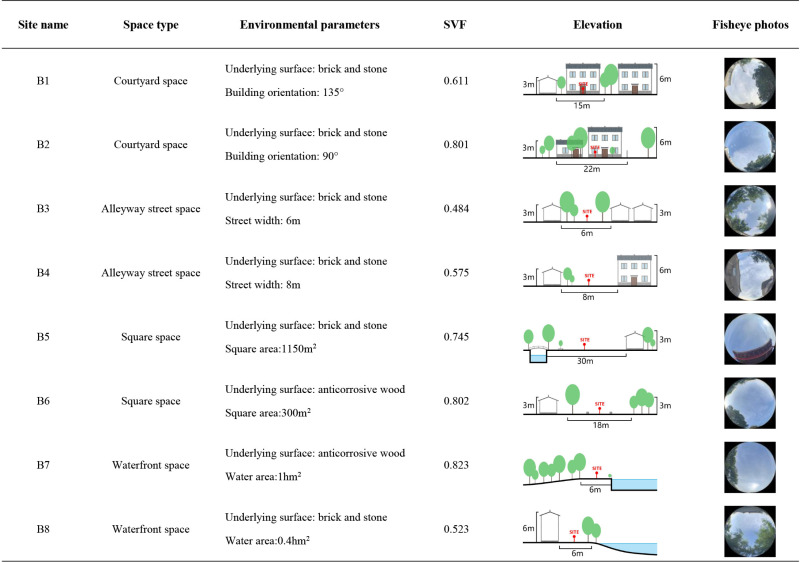
Measurement point settings in Shishanxia Village.

**Fig 5:**
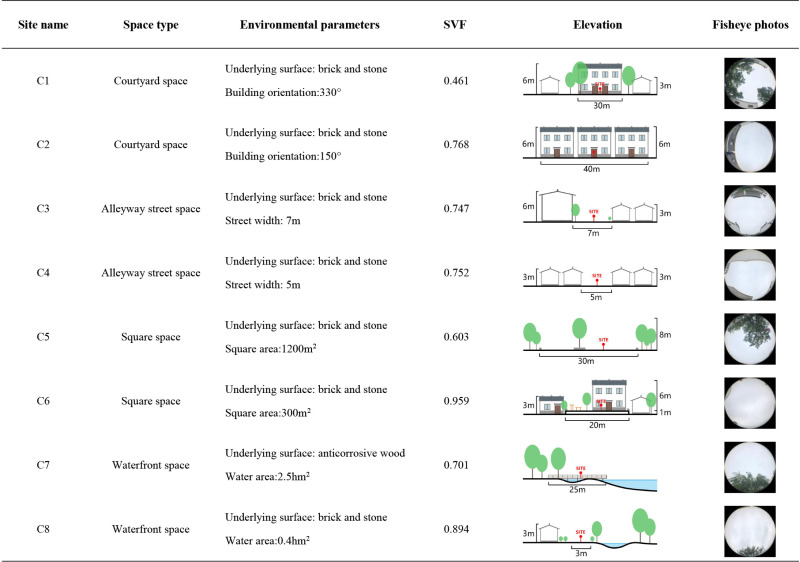
Measurement point settings in Huashu Village.

This study investigates the effects of various spatial configurations and landscape components in villages on microclimate conditions and human thermal comfort. The site is categorized into four distinct spatial types, namely courtyard space, Alleyway space, waterfront space, and square space, based on the external landscape pattern and internal spatial texture of the villages. Each spatial type is accompanied by a corresponding control group. A total of eight measurement points are strategically positioned in each village (Fig 2), as outlined in Figs 3–5.

In the traditional villages characterized by intricate spatial composition, the wind and thermal conditions are subject to the influence of various factors, including street orientation, building arrangement, and vegetation clusters. By factoring in the variables of building characteristics, vegetation distribution, water bodies, underlying surface materials, and sky view factor, a more comprehensive depiction of the spatial configuration, shading conditions, and degree of openness at each measurement point can be achieved. The sky view factor, a crucial geometric parameter in the study of urban heat island phenomena and thermal comfort, serves as a valuable metric for assessing solar radiation and thermal environment [38,39]. Ranging from 0 to 1, the sky view factor value reflects the extent of spatial openness at a given measurement point, with higher values indicating greater openness and vice versa [39,40]. In this study, fisheye images were captured at a height of 1.2 m above ground level at each measurement point, and subsequently, the sky view factor values were computed using the RayMan software [12], as presented in Figs 3–5.

### 2.3 Numerical simulation method

**Fig 6:**
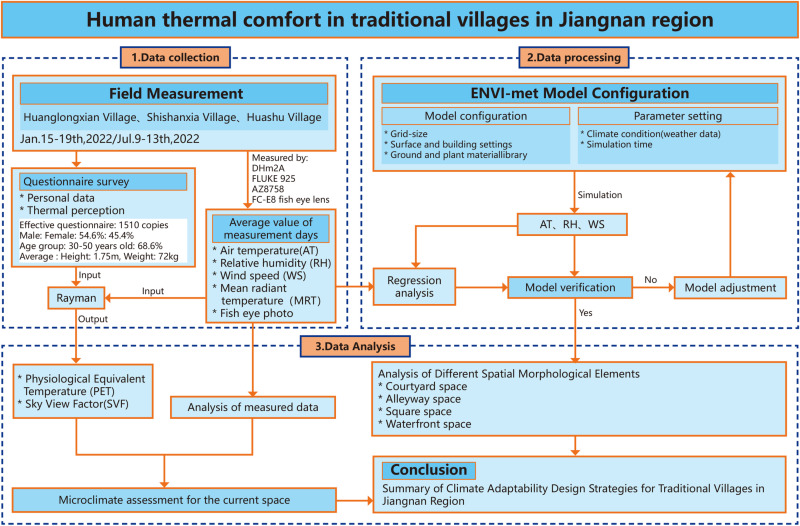
Technology roadmap.

This study employs a combination of field measurement and numerical simulation techniques, as depicted in Fig 6. Firstly, typical meteorological days in winter and summer were selected to conduct field measurements of microclimate factors and thermal comfort questionnaire surveys on the research subjects. Secondly, we conducted on-site random interviews with tourists and villagers, informing them of the main content and purpose of our interview, and asking them if they were willing to accept the questionnaire survey. After obtaining their verbal consent, we provided the questionnaire survey form, which was filled out by the respondents themselves. For minors, we obtained their parents’ consent and conducted a questionnaire survey under their supervision. On this basis, the measured meteorological parameters and human body parameters obtained from questionnaire surveys are input into RayMan software to calculate the physiological equivalent temperature value (PET) for objective assessment of human thermal comfort; At the same time, based on the subjective questionnaire survey results, a subjective analysis of human thermal comfort is conducted to obtain a scientifically authoritative evaluation of the thermal comfort of village public spaces through a combination of subjective and objective methods. Thirdly, through ENVI-met numerical simulations of different spatial types, the microclimate environment of public spaces is visually presented, and the differences and impact patterns of each spatial type control group are analyzed. From an objective perspective, the relationship between traditional village spatial forms and microclimate factors is scientifically analyzed. In addition, before using the ENVI-met model for simulation, its accuracy and applicability must be verified. This study conducted regression analysis between the temperature and humidity measurement data of each measurement point and the ENVI-met simulation data to verify the accuracy of ENVI -met. Many scholars have also used the same method to verify ENVI-met [41–44].

#### 2.3.1 Selection of simulation software.

At present, the most frequently utilized simulation software for environmental microclimate includes CFD and ENVI-met. CFD is predominantly employed for simulating wind environments, whereas ENVI-met stands as the prevailing method for microclimate simulation, with its accuracy having been substantiated across diverse climatic conditions in recent years [45–47]. Therefore, this study opts for the utilization of ENVI-met software to conduct the research, as it enables a more comprehensive depiction of the village’s climatic environment. Specifically, the data for January 17 and July 12 are selected due to their proximity to the average meteorological data over a span of 5 d. The input meteorological parameters are derived from the nearest meteorological station, and the primary verification period spans from 8:00–17:00. However, in order to mitigate potential errors, the total simulation duration is set at 24 h. In the ENVI-met simulation, the parameter values for winter and summer are presented in Table 2.

**Table 2:** Parameter settings in ENVI-met simulation.

Type	Parameters	Value or setting
Site	Location	Nanjing
	Latitude and longitude	32.05°N, 118.78°E
Model domain and grids	Number of grids-X, grids-Y, grids-Z	50 × 50 × 25
	Size of grid cell in meter (meter) (x, y, z)	2 × 2 × 2
Simulation time	Simulation days	Jan.17th,2022/Jul.12th,2022
	Initial time	0
	Duration (h)	24
Meteorological boundaryconditions	Surface roughness	0.1
	Humidity in 2500m	8
	Wind speed at 10m (m/s)	2
	Wind direction (◦)	Meteorological Data

#### 2.3.2 Evaluation index and method of human thermal comfort.

Human thermal comfort is an evaluative assessment of the thermal environment surrounding individuals, as perceived subjectively by humans [5]. Various indicators are commonly employed to evaluate human thermal comfort, including WGBT [48], SET [49], PMV [50,51], PET [42] and UTCI [52,53]. Among these indicators, PET and UTCI have gained widespread usage and have been extensively evaluated [42,54–56]. PET, introduced in 1999, is based on the Munich human heat balance model known as MEMI. It takes into account not only external objective factors such as air temperature, relative humidity, wind speed, and solar radiation intensity, but also internal physiological factors of the human body, including clothing resistance and metabolic rate [57]. However, UTCI does not fully account for the influence of clothing resistance and exhibits certain inaccuracies [58]. In this study, PET is employed as the indicator to quantify outdoor human thermal comfort, with its value calculated using RayMan software [10,58,59].

This study employs physiological equivalent temperature (PET) as an indicator for quantifying human thermal comfort. The PET values for each measurement point between 8:00 and 17:00 are derived by inputting the respective measurement time, latitude, longitude, and altitude, as well as the climatic ((which included the temperature, humidity, wind speed, and mean radiation temperature (T_mrt_)), and human factors specific to each measurement period, into the RayMan software.

For outdoor measurements, the mean radiant temperature can be obtained by approximation from the black globe temperature, air temperature, and wind speed, The calculation formulas are (1):


$$T_{mrt}={[{\left(Tg+273\right)}^{4}+\frac{1.10*10^{8}{Va}^{0.6}}{ƐD^{0.4}}(Tg-Tarbrack}^{\frac{1}{4}}-273$$ (1)

where T_mrt_ is the mean radiant temperature; T_g_ is the black globe temperature (°C); T_a_ is the air temperature (°C); V_a_ is the wind speed (m/s); D is the globe diameter (0.05 m in this study), and Ɛ is the emissivity (0.95 for a black globe).

Drawing upon the findings of a questionnaire survey, the human factors are defined as those of a male individual with a height of 1.75 m, a weight of 72 kg, and an age of 40. The average clothing resistance is set at 0.44 clo during summer and 1.38 clo during winter [44,60]. Given that the respondents are predominantly in a standing or walking posture, the metabolic rate is established as 80W/m[44].

In addition, considering that the PET values, which signify the level of human thermal comfort, vary across different regions, this study refers to the research findings of Chinese scholar Zheng Youfei et al. [61] to determine the physiological equivalent temperature employed in the Nanjing area, as presented in Table 3, for the purpose of analysis.

**Table 3:** Classification of physiological equivalent temperature levels in Nanjing area.

Physiological equivalent temperature/◦C	Human body	Physical stress levels
＜4	Very cold	Extreme cold stress response
≥4-8	Cold	Strong cold Stress response
≥8-13	Cool	Medium cold stress response
≥13-18	Slightly cool	Mild cold stress
≥18-23	Comfort	Non-thermal stress response
≥23-29	Slightly Warm	Mild heat stress
≥29-35	Warm	Medium heat stress response
≥35-41	Hot	Strong heat stress response
≥41	Very hot	Extreme heat stress response

## 3. Results and analysis

### 3.1 Results and analysis of microclimate measurement

#### 3.1.1 Courtyard space.

**Fig 7:**
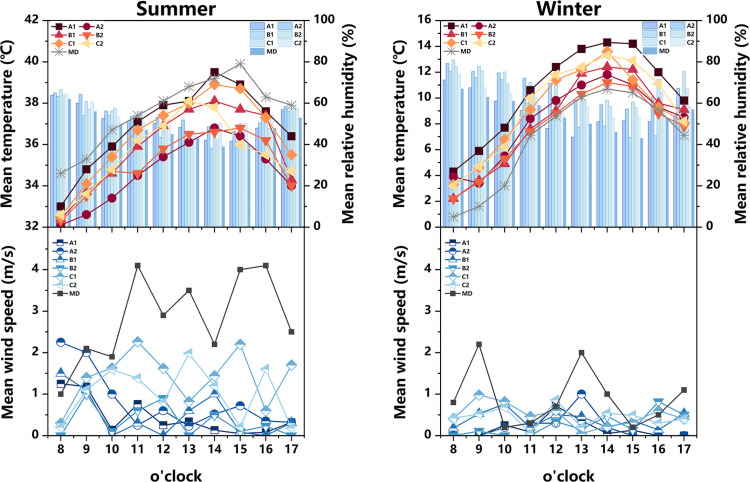
Measured air temperature, relative humidity, and average wind speed at different measurement points in the courtyard space in summer and winter (MD stands for Meteorological Data).

The measured and meteorological data of each measurement point in the courtyard space during the summer and winter seasons are depicted in Fig 7. During the summer, the air temperature at each measurement point exhibits a convex-shaped distribution, with an overall temperature lower than the meteorological value (The MD in Fig 7 represents meteorological data). Notably, the heating rate at measurement point A1 is the most rapid, commencing at 8:00 and peaking at 14:00. The air temperature at measurement points A2 and B2 remains relatively stable, with an average temperature at measurement points B2 (35.1°C) lower than that of other measurement points. Regarding relative humidity, the values at each measurement point demonstrate a pattern of initial decline followed by an increase. Between 8:00 and 14:00, as the sun’s altitude rises, water vapor evaporates from the air and vegetation, leading to a sharp decrease in relative humidity. The average distribution of wind speed at each measurement point exhibits irregularity. The range of wind speed variation is greatest at measurement points C1 and C2, while it is smallest at measurement point B1. During the winter, the air temperature at each measurement point follows a similar trend, with an overall temperature higher than the meteorological value. Notably, the air temperature at measurement points A1 (14.3°C), C1 (13.6°C), and C2 (13.3°C) is relatively high, reaching its peak at 14:00. In terms of relative humidity, measurement points A2 and B2 exhibit the highest values, while measurement point A1 (43.6%) displays the lowest relative humidity, which is 6% lower than the meteorological value at 13:00. The average variation in wind speed at each measurement point is weak, with minimal gap. Additionally, based on the sky view factor presented in Figs 3–5 , the SVF values follow the order A1 > A2 > B2 > C2 > B1 > C1, indicating that measurement point A1 offers the most favorable sky view, indicating that measurement point A1 (0.928) offers the most favorable sky view, the space receives a lot of solar radiation, which can easily cause local high temperatures in summer.Although the SVF values of measurement point A2 (0.817), B2 (0.801), and C2 (0.786) are similar, due to point A2 and B2 proximity to water and lush vegetation, while measurement point C2 only has a small shrub, the building facade and underlying surface absorb and release solar radiation, resulting in an overall high temperature.

Based on the aforementioned analysis, it is observed that measurement points A1 and A2 represent semi-enclosed courtyard spaces, encompassed by buildings on three sides while remaining open on the southern side. Notably, measurement point A1 exhibits a capacious courtyard area devoid of obstructions, thereby allowing for a significant expanse exposed to direct sunlight. Therefore, the air temperature in this space rises rapidly, resulting in higher temperatures during both summer and winter seasons. This phenomenon serves as the primary catalyst for its marginally inferior thermal environment during the summer months. Conversely, measurement point A2 features a greater abundance of vegetation in its premises with evident cooling and humidifying effects. Thus, this location experiences lower temperatures and higher humidity levels during the summer season. Measurement point B1, on the other hand, pertains to a closed courtyard space, encircled by buildings on all four sides. This configuration impedes the circulation of air, thereby yielding minimal fluctuations in wind speed during the summer season. Conversely, measurement point B2 faces a body of water on its northern side, resulting in higher relative humidity levels compared to the other measurement points during both summer and winter seasons. Finally, measurement points C1 and C2 are characterized by buildings flanking their northern and southern sides, while the eastern and western sides remain devoid of structures or vegetation. This arrangement facilitates the formation of ventilation corridors, thereby resulting in evident variations in wind speed.

#### 3.1.2 Alleyway space.

**Fig 8:**
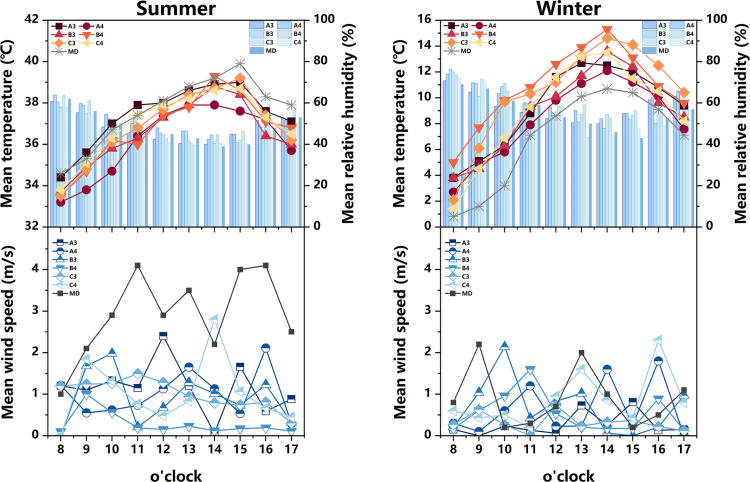
Measured air temperature, relative humidity, and average wind speed at different measurement points in the alleyway space in summer and winter (MD stands for Meteorological Data).

The measured and meteorological data of each measurement point in the Alleyway space during summer and winter are depicted in Fig 8. During summer, the majority of measurement points experience a heating phase from 8:00–14:00, followed by a gradual cooling trend after 14:00. Notably, measurement point A3 exhibits the most rapid heating rate, reaching its peak value(38.6°C) at 15:00, and similarly, it demonstrates the swiftest cooling rate. Concerning relative humidity, measurement points A4, B3, and C3 consistently exhibit higher levels compared to other measurement points throughout most time periods, while the relative humidity fluctuation range at measurement point C4 remains relatively small. Regarding average wind speed, each measurement point displays a significant range of wind speed variations, with measurement point B4 demonstrating the most stable wind speed changes comparatively. During winter, the air temperature at each measurement point surpasses that of the meteorological station. Measurement points B4 and C3 consistently exhibit higher air temperature values, peaking at 14:00. At this juncture, the air temperature at measurement point B4 exceeds the meteorological value by 4.6°C. In terms of relative humidity, the lowest levels are observed at 14:00, with measurement point C4(44.4%) experiencing the lowest relative humidity during this time. As for average wind speed, the fluctuation of average wind speed in the Alleyway space is significant for each measurement point, displaying an irregular distribution. In addition, when considering Figs 3–5 , it becomes evident that measurement point A3(0.934) exhibits the widest street and the most optimal sky view, while measurement point B3(0.484) exhibits the narrowest space and the least optimal sky view. The measurement point A3 with more open space has a larger exposure area in summer, resulting in a higher average temperature. In addition, although the SVF value of measurement point A4 (0.643) is greater than that of measurement point B3, which receives more solar radiation, the average temperature is lower than that of measurement point B3 because its underlying surface is made of white bricks, while measurement point B3 is made of dark gray bricks, which have stronger heat absorption and release properties towards solar radiation.

Generally speaking, the influence of building layout and landscape elements on Alleyway space is of paramount importance. It is worth noting that measurement point A3, featuring the widest street and the most extensive exposure to direct sunlight, exhibits a higher air temperature during the summer months compared to other measurement points. On the other hand, measurement points B4 and C3 find themselves enveloped by a dense cluster of buildings. The cement facade and brick paving of these structures effectively absorb solar radiation and possess commendable heat dissipation properties, thereby providing a thermal insulation effect during the winter season. Therefore, the winter temperature at these measurement points surpasses that of their counterparts. In contrast, measurement points A4 and B3 harbor a profusion of diverse plant species in their premises. These plants, with their evident heating and humidifying effects, contribute to a relatively higher level of relative humidity. The relative humidity at measurement points C4 and B4, however, remains relatively stable. The former is flanked by buildings on both the eastern and western sides, with an absence of plants in its vicinity. The latter is situated at the heart of a street oriented in the southeast-northwest direction. The buildings on either side obstruct the flow of summer and winter winds, and the scarcity of plants in its surroundings further exacerbates this impediment. Moreover, it is worth noting that the arrangement of buildings in the Alleyway space facilitates the formation of ventilation corridors. Hence, the fluctuation range of average wind speed in this domain surpasses that of the courtyard space.

#### 3.1.3 Square space.

**Fig 9:**
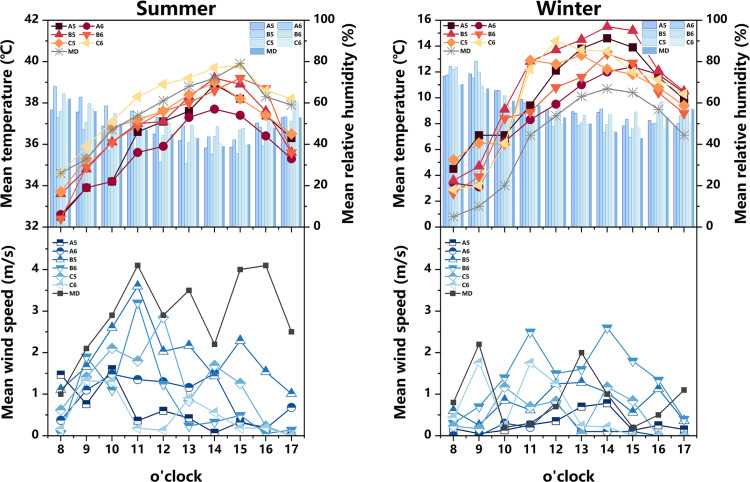
Measured air temperature, relative humidity, and average wind speed at different measurement points in the square space in summer and winter (MD stands for Meteorological Data).

The measured and meteorological data of each measurement point in the square area during the summer and winter seasons are presented in Fig 9. During the summer season, a rapid increase in temperature is observed at measurement point C6 from 8:00–15:00, reaching its peak (39.7°C), followed by a gradual decrease. The air temperature at measurement point A6 exhibits the lowest average value (35.63°C), which is 2.5°C lower than the meteorological value at 15:00. The relative humidity graph reveals that the overall relative humidity at measurement points A6(53.46%) and C5(54.34%) is higher compared to other measurement points. Measurement point B5 exhibits the smallest fluctuation range in relative humidity, with the lowest mean value. Regarding the average wind speed, significant fluctuations are observed in the average wind speed values at each measurement point. Notably, measurement points A5 and C6 exhibit relatively smaller wind speed fluctuations. During the winter season, the air temperature at each measurement point is higher than that recorded at the meteorological station. The peak air temperature at each measurement point occurs at 14:00, with measurement point B5 (39.7°C) exhibiting the highest mean value, while measurement point A6 (12°C) exhibits the lowest. In terms of relative humidity, the overall relative humidity at each measurement point surpasses that of the meteorological station, with measurement point C6(53.4%) exhibiting the lowest mean value. The average wind speed at each measurement point demonstrates irregularity and randomness. The highest average wind speed value is observed at measurement point B6 (1.414m/s), whereas the lowest is recorded at measurement point A5 (0.16m/s). Analysis of the sky view factor in Figs 3–5 reveals that measurement point C6 (0.959) possesses the most expansive and unobstructed space, providing the best sky view, and the exposure area is larger in summer, so the average temperature in summer is higher. Although the SVF value of measuring point A6 (0.802) is higher than that of measuring point C5 (0.603), due to its proximity to water, its average temperature is the lowest under the cooling and humidification effect of the water.

According to the aforementioned findings, it is observed that measurement point B5 possesses a large site area, yet exhibits a scarcity of vegetation. Therefore, a significant portion of this area is exposed to direct sunlight, resulting in higher temperatures and a narrower range of relative humidity fluctuations. The building envelope and brick paving at measurement point C6 demonstrate a propensity for solar radiation absorption, thereby facilitating a rapid heating rate and generally higher site temperatures. This phenomenon serves as the primary catalyst for the slightly inferior thermal conditions experienced during the summer months. Measurement points A6 and C5, in close proximity to a water body and enveloped by luxuriant vegetation with intricate layers, benefit from the stabilizing influence of transpiration from both the water body and plants. Thus, these points exhibit the highest mean values of relative humidity. In comparison to the aforementioned three spatial categories, leisure spaces feature more expansive open areas, thereby exposing larger surface areas to direct sunlight and subsequently resulting in relatively higher temperatures. In addition, the majority of leisure spaces are adorned with an abundance of vegetation, which facilitates a favorable ventilation effect, leading to more pronounced fluctuations in instantaneous wind speed.

#### 3.1.4 Waterfront Space.

**Fig 10:**
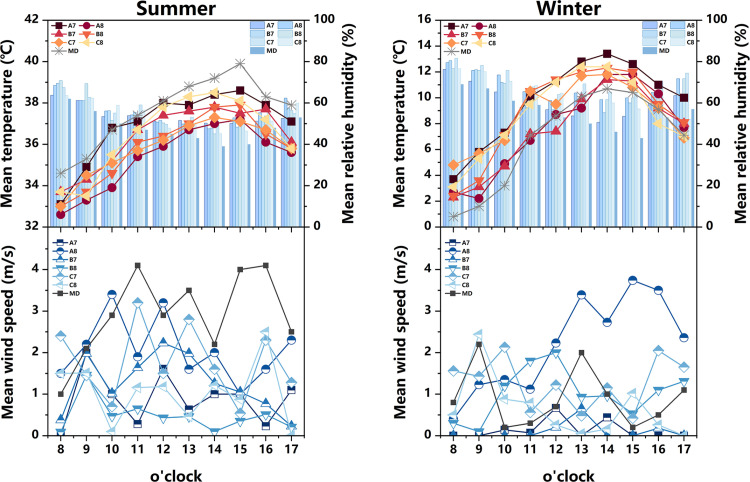
Measured air temperature, relative humidity, and average wind speed at different measurement points in the waterfront space in summer and winter (MD stands for Meteorological Data).

The measured and meteorological data of each measurement point in the waterfront space during summer and winter are depicted in Fig 10. During the summer season, the temperature variations observed at each measurement point exhibit a similar trend, consistently lower than the corresponding meteorological values. Notably, measurement point A7 (36.98°C) demonstrates a generally higher air temperature, while measurement point A8 (35.37°C) exhibits a lower temperature. Regarding relative humidity, the values recorded at each measurement point surpass the meteorological values, albeit with minimal differences. The wind speed experiences significant fluctuations across all measurement points, with the most significant range observed at measurement points A8(2.07m/s) and C7(1.77m/s). In the winter season, the majority of measurement points display a rising and subsequently declining trend in air temperature. Prior to 14:00, the temperature exhibits an overall increase, followed by a sharp decline thereafter. Measurement points A8 (7.6°C) and B7 (7.45°C) record relatively lower air temperatures. Concerning relative humidity, the mean values at measurement points A8(68.09%) and C7 (71.1%) are the highest. Notably, 14:00 represents the daily minimum for relative humidity. At this juncture, measurement point C7 (65.3%) records the highest relative humidity, surpassing the meteorological value by 19.3%. Conversely, measurement point A7 (50.6%) records a lower relative humidity, yet still 4.6% higher than the meteorological value. The average wind speed fluctuations during winter are generally smaller compared to those observed during summer. According to the sky view factor in Figs 3–5, the SVF value of measuring point A8 (0.775) is higher than that of measuring points A7 (0.714), B8 (0.523), and C7 (0.701), but the buildings on its south side have the effect of blocking solar radiation, resulting in the lowest average temperature.

In summary, measurement points A7 and C8 exhibit smaller water areas and lack building obstructions on their southern side, leading to generally higher temperatures. Conversely, measurement points A8 and C7 feature larger water areas surrounded by abundant vegetation. The combined transpiration effects of the water bodies and plants contribute to increased relative humidity and greater wind speed fluctuations. The waterfront space, influenced by the cooling and humidifying properties of the adjacent water body, demonstrates a higher average relative humidity compared to the other three space types, while maintaining a lower average temperature.

### 3.2 Results and analysis of physiological equivalent temperature

**Fig 11:**
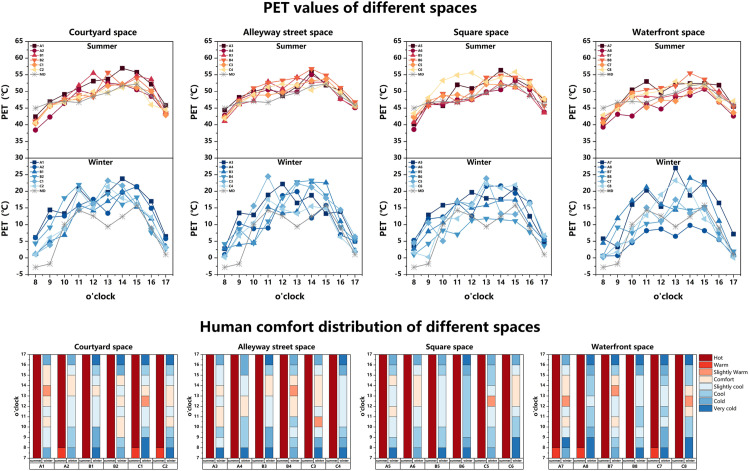
PET values of different spaces in summer and winter and Human comfort distribution of different spaces in summer and winter.

As depicted in Fig 11, the PET values of each spatial domain exhibit more pronounced fluctuations during the winter season. Concerning the thermal comfort experienced in summer, the PET values recorded at each measurement point range from 38.4–57°C, displaying an overall distribution characterized by a convex shape. The difference in PET values among the various spaces is not significant during the summer period, primarily categorized as “very hot” and “hot,” with the former being more prevalent. Notably, both the Alleyway space and square space consistently fall under the classification of “very hot,” indicating their unsuitability for prolonged summer sojourn. Conversely, during winter, the PET values measured at each point range from 0.1–27°C, exhibiting a more evident range of variation comprising five distinct categories of human thermal comfort perception: “very cold,” “cold,” “cool,” “slightly cool,” “comfortable,” and “slightly warm.” The increment of solar radiation initiates the emergence of human comfort at 10:00. Between 12:00 and 15:00, the courtyard space and Alleyway space predominantly attain a state of “comfortable” thermal comfort, while the square space and waterfront space primarily appear as “slightly cool” and “cool”.

From a spatial perspective, in the courtyard space, point A1 has the highest average PET temperature in summer (50.7°C), which is due to the large area of direct sunlight and high temperature in the space, resulting in thermal discomfort. The summer PET mean of measurement point A2, which has a small space and more vegetation, is the lowest (47.5°C). In the alleyway space, The winter PET average of measuring point C4 is the lowest (10.1 °C), because its streets and alleys are oriented in a north-south direction, and the space is prone to forming ventilation corridors with high wind speeds, which exacerbates the discomfort of the human body. In the square space, the summer PET average of measurement point C8 is the highest (51.9°C), which is due to the absorption and heat release of solar radiation by the building skin and brick flooring, resulting in high space temperature and poor thermal comfort. In the waterfront space, the measurement point C7 with the largest water area has the lowest winter PET mean (8.75°C), indicating that the cooling and humidification effect of water has a significant impact on thermal comfort.

Upon comprehensive comparison of the four spatial types, during summer the courtyard space appears to be relatively hotter, while during winter it is the most comfortable, thereby exhibiting the most favorable conditions for human thermal comfort among the four spatial types, rendering it suitable for extended stays. Conversely, the Alleyway space exhibits inadequate human thermal comfort during summer, while the square space experiences hot conditions during summer and cold conditions during winter, thereby displaying the least favorable human thermal comfort and unsuitability for prolonged stays. The waterfront space offers relatively better thermal comfort during summer but deteriorates during winter, rendering it unsuitable for extended stays during the winter season.

### 3.3 Analysis of different space types and climate effects

Chinese traditional villages exhibit diverse spatial typologies with distinct functionalities influenced by various factors such as buildings, plants, and water bodies. This paper analyzes the microclimate effects of each space type in three villages using measured data and ENVI-met numerical simulation. The aim is to provide scientific and reasonable suggestions for climate-adaptive design in rural areas.

**Fig 12:**
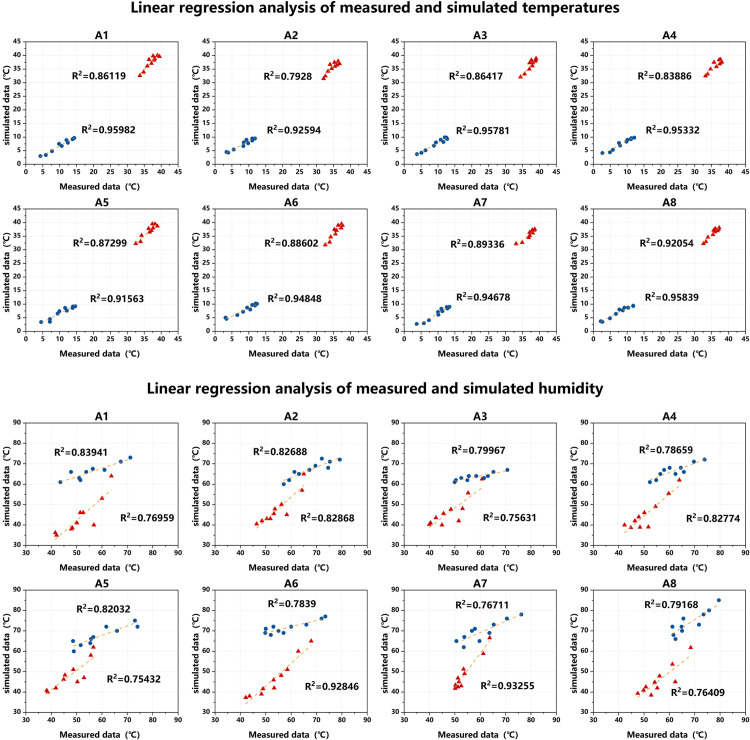
Linear regression analysis of measured and simulated at different measurement points in Huanglongxian Village in summer and winter.

Before employing the ENVI-met model for simulation, it is crucial to verify its accuracy and applicability. Thus, this study regresses the measured data of AT and RH at each measurement point with the simulated data from ENVI-met to validate its accuracy. Taking Huanglongxian village as an example (Fig 12), the R^2^ values for temperature and humidity simulation data compared to measured values exceed 0.75 in both winter and summer. Previous studies [62,63] indicate acceptable accuracy of the simulation data, with differences attributed to variations in material properties [64,65]. In conclusion, the verification results demonstrate that the ENVI-met model accurately reflects the conditions at each measurement point in the village, validating the use of its data for the current analysis.

#### 3.3.1 Courtyard space.

The three villages selected in this paper possess the quintessential attributes of traditional villages in southern China, exhibiting a spatial arrangement characterized by a “house-courtyard-courtyard group” configuration, predominantly featuring semi-enclosed courtyards. Serving as the primary spatial element in the village, the buildings constitute the fundamental units of the courtyard space. The orientation, height, density, and spatial composition of these structures represent pivotal factors that exert a profound influence on the microclimate.

**Fig 13:**
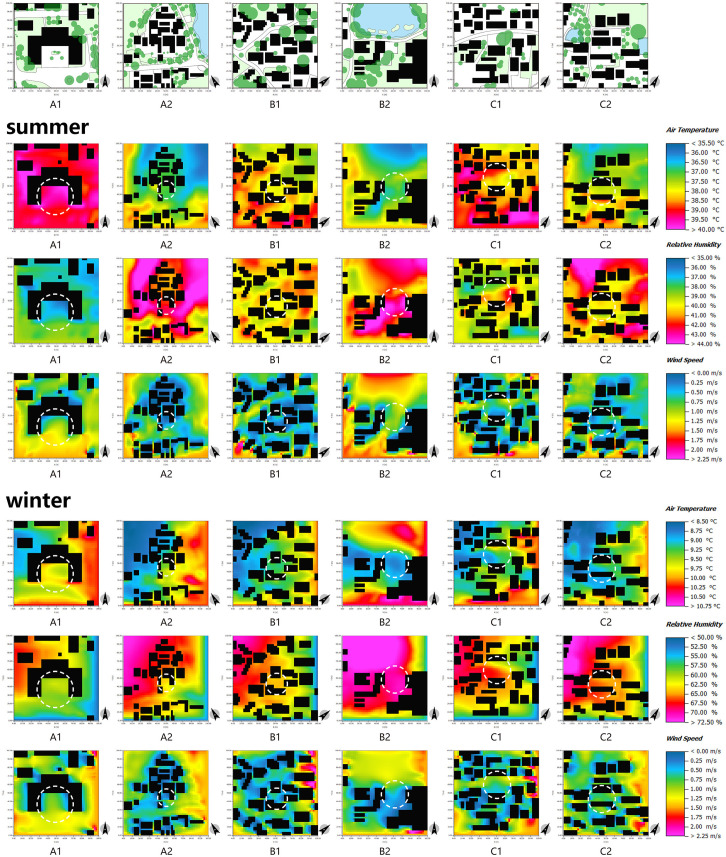
ENVI-met simulation of different measurement points in the courtyard space in summer and winter.

The orientation of the buildings significantly impacts the absorption of solar radiation in the courtyard space, thereby constituting the principal determinant of the spatial thermal environment. Analysis of the temperature simulation map of the courtyard space during summer and winter (Fig 13) reveals that measurement point A1 experiences higher temperatures during both seasons, while measurement point B2 exhibits the lowest temperatures. This discrepancy arises from the south-facing orientation of measurement point A1 and the east-facing orientation of measurement point B2. The south-facing buildings facilitate the acquisition of a greater amount of solar radiation, thereby creating a comfortable thermal environment during winter. However, during summer, this orientation may result in excessively high space temperatures, which can be mitigated through the strategic incorporation of vegetation to reduce the ambient temperature.

**Fig 14:**
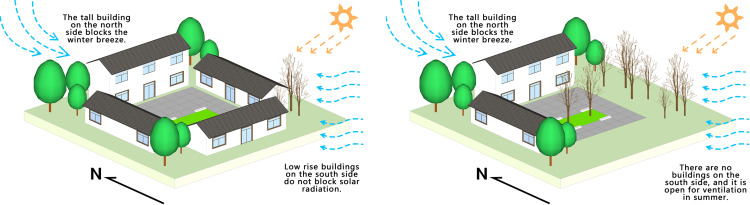
The influence of courtyard space building height on thermal environment.

In the construction of villages in southern China, it is recommended to implement a spatial arrangement characterized by lower building heights in the southeast and higher building heights in the northwest (Fig 14). This particular layout is advantageous in enhancing the comfort of the wind environment, particularly under the influence of monsoon patterns. The measurement points A1 and A2 pertain to courtyard spaces enclosed by buildings on three sides. The buildings situated on the eastern and western sides are single-story structures. Notably, the building adjacent to the northern side of measurement point A1 is also single-story, while the building neighboring measurement point A2 is two stories high. By referring to Fig 13, it becomes evident that the wind speed at measurement point A2 is comparatively lower. This can be attributed to the presence of a taller building on its northwest side, which effectively acts as a barrier against the northwest monsoon. Therefore, this wind barrier not only fulfills the requirements of thermal insulation and temperature regulation during winter but also mitigates the wind speed in the courtyard space. Conversely, the construction of excessively tall buildings on the southeast side obstructs solar radiation, resulting in reduced internal temperatures. In addition, it hinders the entry of summer winds into the courtyard, as exemplified by measurement point C1, characterized by low winter temperatures, reduced summer wind speeds, and higher temperatures. Such a configuration leads to a sense of stuffiness and inadequate thermal comfort.

The air circulation in courtyard spaces is influenced by the density and spatial arrangement of buildings. Fig 13 provides visual evidence that courtyard spaces characterized by high building density, such as measurement points A2, B1, C1, and C2, exhibit a greater degree of spatial enclosure. Therefore, the wind speed of the external monsoon entering the village interior is weakened, resulting in lower wind speeds in these areas. The courtyard spaces with high building density serve the purpose of temperature regulation and wind prevention, a notion that has been acknowledged by other researchers in the field [66].

#### 3.3.2 Alleyway space.

Alleyway space, serving as the structural framework and vital component of traditional villages, fulfills both transportation and spatial node connection functions. Its spatial arrangement significantly influences the overall spatial texture and form of the village. In the Alleyway space, the scale and height-width ratio of streets exert a profound impact on the wind and thermal environment of the villages.

**Fig 15:**
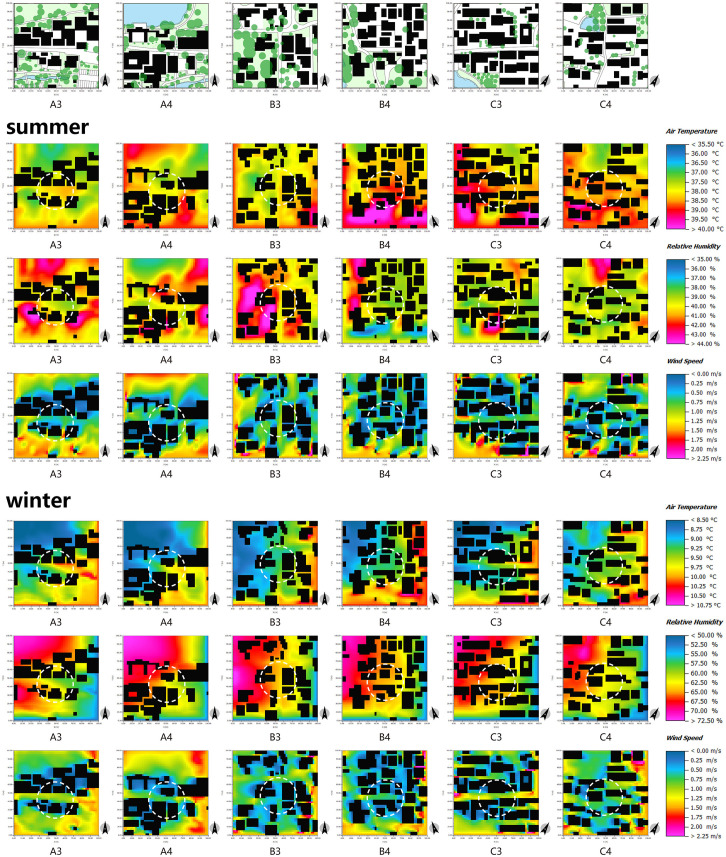
ENVI-met simulation of different measurement points in the alleyway space in summer and winter.

**Fig 16:**
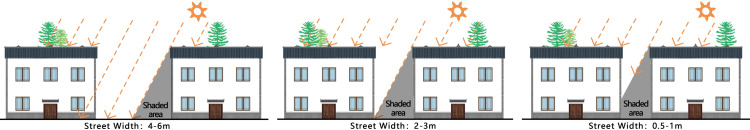
The influence of building height on microclimate in courtyard space.

First of all, the scale of streets directly impacts the wind environment of said streets. As depicted in Fig 15, measurement points A3 and A4 are situated in the central region of the primary thoroughfare, which features a greater width compared to other areas (Figs 3–5). These points are flanked by buildings on both sides, thereby shielding them from counterflow air currents. Therefore, the range of wind speed fluctuations experienced at these points is more pronounced. Secondly, the thermal conditions in street spaces are predominantly influenced by the height-width ratio of the streets themselves (Fig 16). By cross-referencing Figs 3–5 and Fig 15, it becomes apparent that the northern and southern sides of measurement point A3 are occupied by single-story structures, while the street width spans 12 m. Conversely, the northern side of measurement point C3 features a two-story building, while the southern side is occupied by a single-story structure, and the street width measures 7 m. In relative terms, measurement point C3 exhibits a higher height-width ratio, resulting in higher temperatures during summer and reduced temperatures during winter. Thus, the level of human comfort experienced at this point is reduced. In summary, a lower height-width ratio facilitates increased absorption of solar heat radiation by the site, thereby enhancing the thermal conditions in the Alleyway space. This, in turn, causes a temperature differential between the interior and exterior spaces, promoting thermal pressure ventilation and facilitating the exchange of air between the two. Conversely, a higher height-width ratio readily gives rise to a somber environment, leading to decreased temperatures and a sense of oppression among individuals. Accordingly, the level of human comfort experienced in such circumstances is notably suboptimal, a notion that is corroborated by other literature[67].

#### 3.3.3 Square space.

The square space of traditional villages involves a multitude of functions, including recreation, socialization, production activities, product sales, and festival entertainment, all of which exhibit a plethora of morphological characteristics. The square space, owing to its high degree of openness, is significantly influenced by the surrounding plant assemblages and the underlying surface materials, thereby impacting its microclimate.

**Fig 17:**
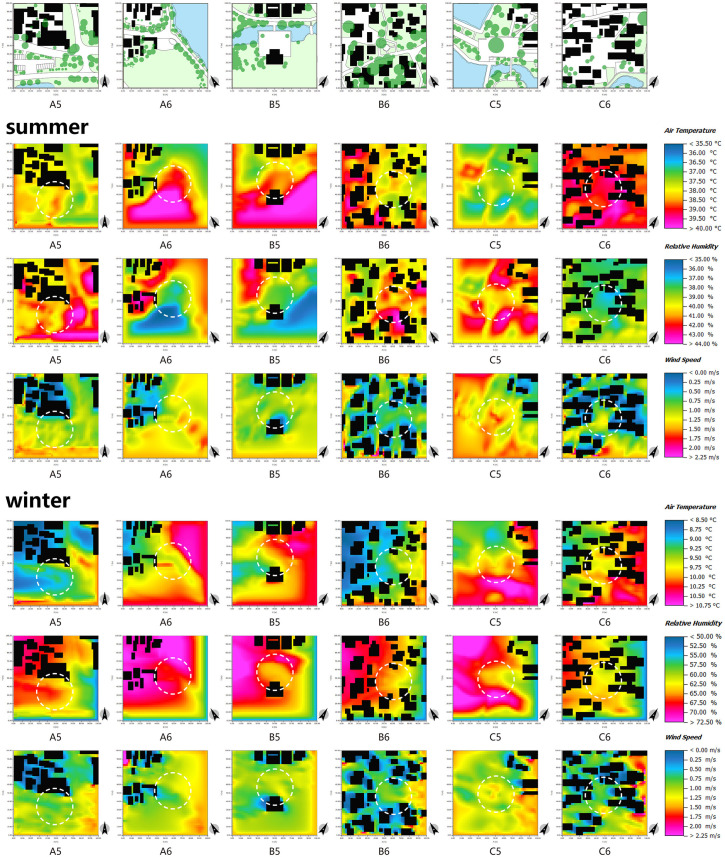
ENVI-met simulation of different measurement points in the square space in summer and winter.

First and foremost, in the square space, the wind and thermal environment are profoundly affected by the presence of plant groups. As depicted in Fig 17, it is notable that measurement point C6 is surrounded by a sparse vegetation arrangement, characterized by an arbor-grass (evergreen) planting mode. Conversely, measurement points A5 and B6 exhibit an abundance of vegetation, exemplifying a multi-layer planting mode comprising arbor-shrub-grass (evergreen) elements. Evidently, the cooling and humidifying effects of the latter two measurement points surpass those of measurement point C6. In addition, in comparison to measurement point B6, measurement point A5 exhibits a lower degree of plant enclosure. While this facilitates solar radiation absorption, it hampers wind speed control, resulting in greater wind speed fluctuations and rendering it unsuitable for prolonged winter stays. Thus, it is apparent that the mode and enclosure degree of plant groups are intricately linked to human thermal comfort. Generally speaking, the arbor-shrub-grass (evergreen) plant combination yields the most favorable cooling and humidifying effects [51]. Additionally, an appropriate enclosure degree enables the regulation of solar radiation and wind speed, thereby modulating the microclimate. During winter, a plant group with a high enclosure degree and abundant layers on the northwest side can serve as a natural wind barrier, affording wind protection and thermal insulation [59,68,69], as exemplified by measurement point B5. Conversely, the southeast direction should predominantly feature deciduous plant groups with a low enclosure degree and transparent structure. These plants effectively obstruct direct sunlight during summer, creating a cool environment. In winter, they readily absorb sunlight, thereby maintaining a comfortable thermal perception.

In addition, in the context of the microclimate of villages, it is crucial to consider the impact of the underlying surface on solar radiation absorption. The traditional villages predominantly feature various materials such as brick stone, concrete, permeable brick, grass brick, and lawn as their underlying surfaces. Specifically, measurement points B5 and C6 are characterized by concrete and brick stone materials. These materials possess a significant specific heat capacity, high thermal conductivity, and an increased heat absorption rate. Therefore, they effectively absorb solar radiation, leading to an increase in the ambient temperature. Moreover, this phenomenon accelerates the evaporation of water in the atmosphere, resulting in reduced environmental humidity. While this characteristic proves advantageous in mitigating the humid and cold environment during winter, it exacerbates the heat sensation in the space and reduces human thermal comfort during summer. On the other hand, measurement point B6 exhibits a wooden paving and lawn as its underlying surface. Permeable brick, grass brick, lawn, and farmland are materials known for their commendable water permeability and low heat absorption rate of solar radiation. These materials exhibit superior temperature stability and offer enhanced thermal comfort. In conclusion, in regions characterized by hot summers and cold winters, such as southern China, it is advisable to opt for underlying surface materials with a limited specific heat capacity, low thermal conductivity, and excellent water permeability.

#### 3.3.4 Waterfront space.

The selection of sites for traditional villages adheres to the principle of “ water first, then village” and “water is superior”, while also considering the spatial arrangement of facing water and having mountains behind. The presence of water bodies, an essential component of traditional villages in southern China, serves not only practical purposes such as living, transportation, and aesthetics, but also plays a significant role in regulating the microclimate. Owing to the high specific heat capacity of water, it undergoes evaporation and absorbs heat from the atmosphere as solar radiation intensifies during the daytime. Therefore, the water vapor content in the air increases, leading to a noticeable cooling and humidifying effect. During the nighttime, the water bodies release the heat accumulated throughout the day, thereby providing a certain degree of thermal insulation. This phenomenon contributes to the creation of an environment characterized by minimal diurnal temperature fluctuations[70].

**Fig 18:**
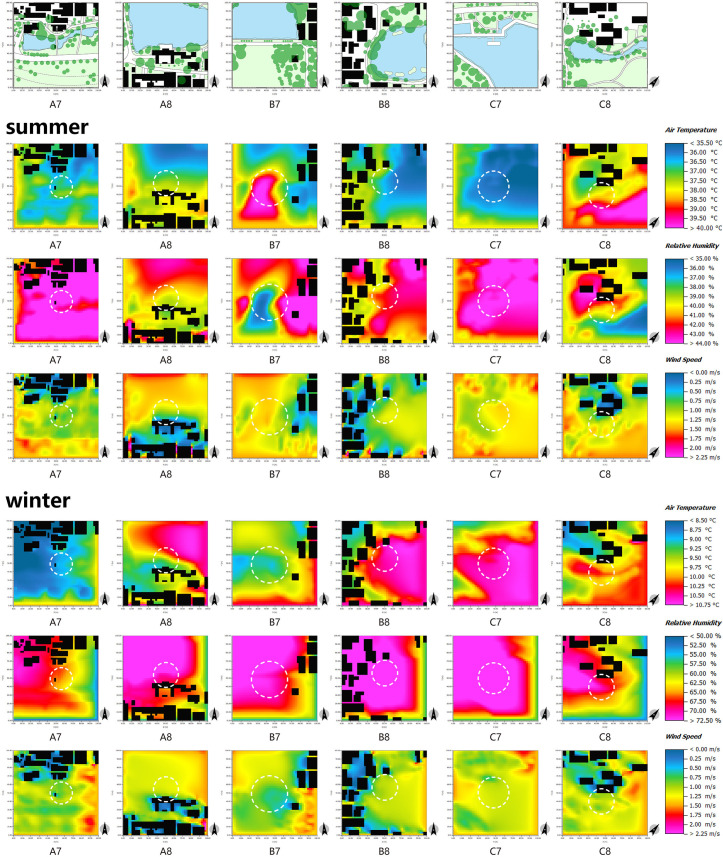
ENVI-met simulation of different measurement points in the waterfront space in summer and winter.

The regulation effects of water area and location on the wind and thermal environment of villages are readily apparent. Upon analysis of Fig 18, it can be observed that expansive water bodies, exemplified by measurement points A8, B7, and C7, exhibit higher local wind speeds owing to their expansive water surfaces and the temperature difference between water and land. In addition, the cooling and humidifying influence of these water bodies significantly reduces human comfort during the winter months. Moreover, areas characterized by buildings obstructing the northwest side and water bodies adorning the southeast side offer optimal thermal comfort, as exemplified by measurement point B8. The buildings on the northwest side effectively shield against winter winds, while the summer winds sweep over the water bodies, introducing large amount of water vapor into the village. This phenomenon is conducive to reducing the temperature and humidity in the village, thereby enhancing human thermal comfort.

## 4. Discussion

**Fig 19:**
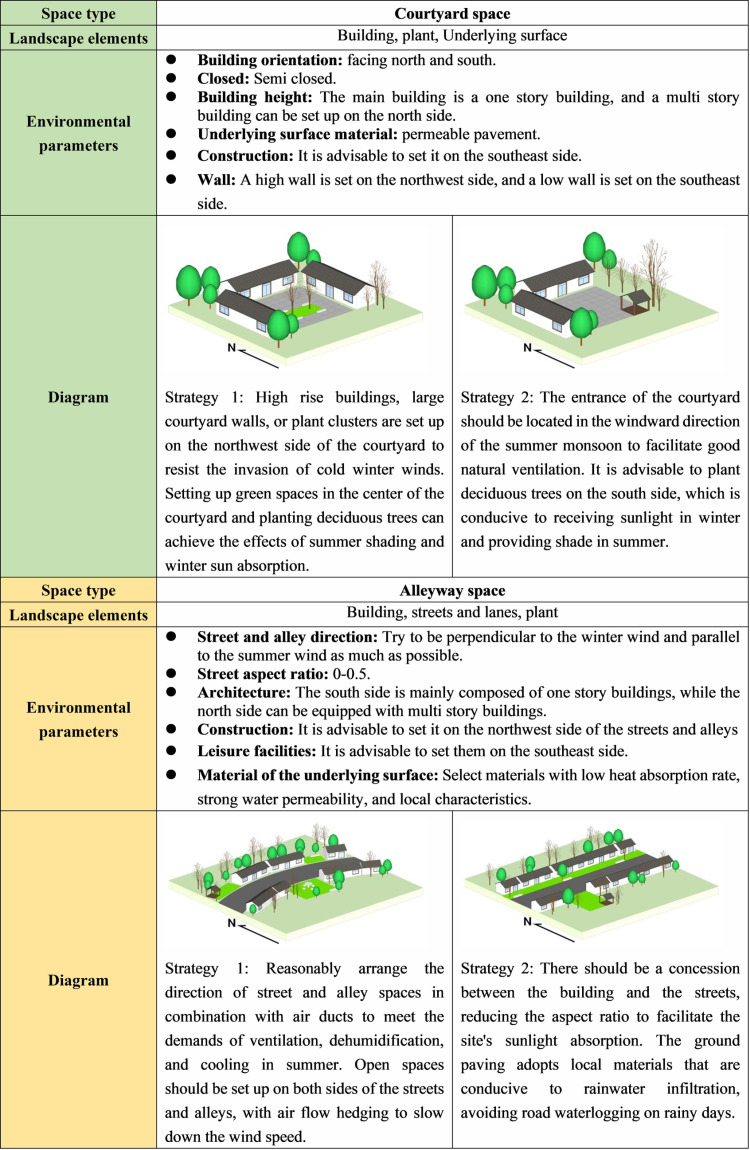
Climate adaptive design of Courtyard space and Alleyway space.

**Fig 20:**
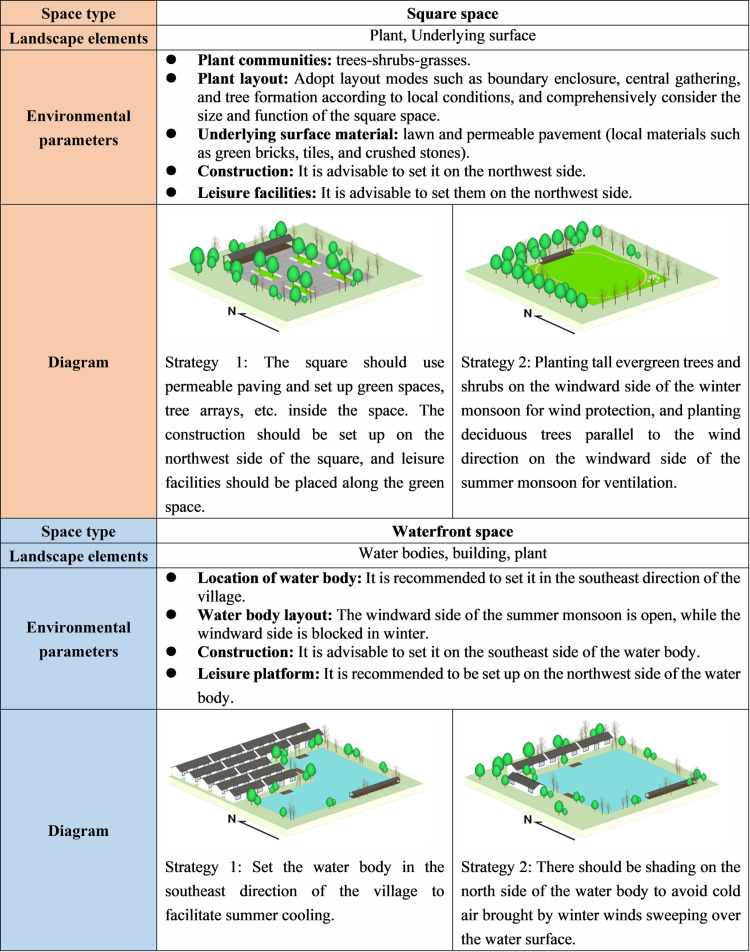
Climate adaptive design of Square space and Waterfront space.

Drawing upon typical traditional villages in southern China as a case study, this study analyzes the relationship between traditional village spatial form and microclimate based on measured data and computer simulation. It analyzes the modes of spatial renewal that enhance climate adaptability in regions with hot summers and cold winters. The study concludes by presenting transformation strategies (Figs 19–20), aiming to offer guidance and inspiration for the construction of environmentally friendly and livable new rural areas in China.

(1) Residential buildings constitute a pivotal facet of the village fabric. In the optimization of courtyard spaces, it is necessary to perpetuate the spatial arrangement and architectural style peculiarities of village courtyards. In the case of aged structures, while adhering to the principle of preserving the old, the height and density of buildings must be judiciously regulated to satisfy the requisites of winter wind protection and solar irradiation, as well as summer ventilation and shading, thereby creating a comfortable and pleasant courtyard space[30,71]. As for new buildings, by devising appropriate building orientations and spatial configurations, they can not only withstand wintry gusts and maintain spatial thermal equilibrium, but also facilitate the formation of ventilation corridors during summer, effectively channeling natural breezes.
(2) In the process of their development, traditional villages have established a relatively comprehensive street spatial pattern. Therefore, when enhancing and renovating the Alleyway space, it is necessary to uphold the original street context. This can be achieved by judiciously regulating the height-width ratio of streets and incorporating local spatial nodes[44]. Accordingly, the control of solar radiation and air circulation is facilitated. Therefore, a delightful and harmonious Alleyway space is created, thereby protecting and perpetuating the original pattern and unique features of village streets.
(3) The presence of water bodies is an indispensable component of traditional villages in southern China. However, a single water body exerts minimal influence on the wind and thermal environment of villages. Conversely, by appropriately configuring the combination and proportion of water bodies with other landscape elements, their cooling and humidifying effects can be significantly enhanced. For instance, the utilization of variations in terrain elevation can give rise to natural water channels, while interconnecting various water bodies in villages can establish a comprehensive water system. This approach serves to accentuate the quintessential characteristics of water towns in southern China.
(4) Traditional villages exhibit typical climate adaptability characteristics in their plant cultivation patterns, which have evolved naturally over an extended period. When optimizing the plant landscape of these villages, it is necessary to select indigenous tree species that can form near-natural plant assemblages comprising trees, shrubs, and grasses[72]. Additionally, a comprehensive assessment of factors such as the degree of enclosure, planting orientation, deciduous-to-evergreen ratio, monsoon wind direction, and the ecological benefits of plant communities must be undertaken to ensure the attainment of microclimate effects, including ventilation, wind resistance, shading, sunshine reception, and humidification.
(5) The selection of underlying surface materials for traditional villages necessitates the consideration of both the physical properties of the materials and their compatibility with the surrounding environment, taking into account the specific spatial context. On one hand, materials with high specific heat capacity and excellent water permeability should be chosen as underlying surface materials for villages to regulate solar radiation and maintain a balanced surface temperature and humidity environment. On the other hand, in conjunction with pattern design, local materials such as brick blocks, gravel, and tiles should be selected to not only enhance the textural richness of the village surface but also maintain consistency with the overall architectural style.

This study approaches the spatial form of traditional villages in the Jiangnan region from the unique perspective of human thermal comfort, providing new ideas and methods for research in related fields. Previous studies have mostly focused on the historical and cultural value, architectural style, and other aspects of traditional villages. This article fills some of the gaps in the research on the relationship between thermal environment and spatial form in this field by quantitatively studying the correlation between thermal comfort indicators and spatial form elements. It provides a more comprehensive theoretical basis for future scholars to study the livability, sustainable development, and other aspects of traditional villages. This interdisciplinary research approach integrates knowledge from multiple disciplines such as architecture, environmental science, and ergonomics, expanding the boundaries of traditional village research and promoting its development towards multidimensional and refined directions.

Although traditional villages in the Jiangnan region have their unique geographical, climatic, and cultural characteristics, the inherent connection between thermal comfort and spatial form is to some extent universal. Other regions can refer to the methods and ideas of this study when planning villages, and optimize the spatial form of villages by combining local climate conditions, topography, and cultural traditions.

In addition, the results of this study have important reference value for current rural development policies. Policy makers can plan the distribution of roads, greenery, and public spaces reasonably based on the demand for thermal comfort when formulating relevant policies, ensuring that the original thermal environment regulation mechanism of traditional villages is not destroyed while meeting the needs of modern life, and promoting the sustainable development of traditional villages.

## 5. Conclusion

This article takes three typical traditional villages in Nanjing as research objects. Based on measured data results and ENVI met numerical simulations, the microclimate effects of courtyard space, street space, square space, and waterfront space in the three villages are analyzed. The thermal comfort PET values are calculated using the Rayman platform, and the differences and impact patterns with each spatial control group are analyzed. From an objective perspective, the relationship between the spatial form of traditional villages in Jiangnan and microclimate factors is scientifically analyzed. Research has found that:

1. The PET value in summer is between 38.4–57°C, and in winter it is between 0.1–27°C. Overall, the thermal comfort in winter is better than that in summer.
2. The variation range of air temperature and relative humidity in the courtyard space is relatively stable, and the thermal environment is greatly affected by the orientation of the building. The courtyard space facing south has a better overall thermal environment.
3. Alleyway spaces are affected by the absorption and reflection of solar radiation by the building facade and underlying surfaces, resulting in generally higher air temperatures than other spaces; Due to the influence of the aspect ratio and orientation of streets and alleys, smaller aspect ratios reduce obstruction between buildings, increase solar radiation area, and ensure air circulation. Therefore, streets and alleys with smaller aspect ratios have better wind and heat environments.
4. The square space is spacious and has the best ventilation effect. The plant conditions and underlying surface materials in the square space have a significant impact on the microclimate of the site. Spaces with high plant canopy density and rich layers have higher relative humidity. Spaces with underlying surfaces made of materials with high specific heat capacity, thermal conductivity, and heat absorption rate have poor thermal comfort in summer.
5. The waterfront space is affected by the cooling and humidifying effects of water evaporation, resulting in lower air temperature and higher relative humidity. The larger the water surface area, the higher the relative humidity and wind speed of the space.

Even though certain results have been achieved through this study, further improvement is still needed. In future research, it is necessary to Investigate more villages, set up more monitoring points, extend the monitoring data time, in order to obtain more and more detailed microclimate data, and draw more universal conclusions. In addition, in future research on climate thermal comfort, our team will further investigate different countries and regions with similar latitude and longitude, and draw universal conclusions to promote the development of international research. We not only hope to provide guidance for the spatial design of traditional villages in the Jiangnan region of China, but also hope to provide new ideas for the spatial design of traditional villages in different regions, and contribute to rural revitalization.

## Supporting information

Supporting information This file contains the supplementary figures and S2 Files. (ZIP)
