# Supplementary material for: A Study on the spatial form of traditional villages in jiangnan region of china from the perspective of human thermal comfort: A case study of nanjing, jiangsu province
Source: PLoS One. 2025 May 9;20(5):e0323252. doi: 10.1371/journal.pone.0323252 (PMC12064036; doi:10.1371/journal.pone.0323252)
Supplement: Supporting information — (ZIP) [file pone.0323252.s001.zip › Supporting Information/S2 File. for details Questionnaire used for data collection.docx]

**Human Thermal Comfort Survey Questionnaire**

**【Basic information】**

1. **Gender**：□Male □Female
2. **Age：**
3. **Height：** □≤140cm □141—150cm □151—160cm □161—170cm

□171—180cm □181cm —190cm □191cm —200cm □≥201cm

1. **Weight：** KG
2. **Current outdoor stay time：**□ 0.5-1h □1-2h □ 2-3 h □3-4h □≥4h

**7. Current behavior pattern：**□Standing □Sitting □Walking

**8. Current attire：**

**Top：** □Long sleeved □short sleeved □sleeveless □Jacket □cotton coat □sweater □down jacket □thin coat □nylon coat □Other：

**pants：** □Long pants □mid pants □shorts □long skirt □short skirt □Cotton pants □woolen pants □jeans □casual pants □leather pants □Other：

**Shoes：** □Sandals □sneakers □mesh sports shoes □Leather Shoes □Boots □Cotton Shoes □Other：

**Hat：** □Yes □No

**【Investigation on Human Thermal Comfort】**

1. **What is your overall feeling about the current outdoor environment :**

□Very comfortable □Quite comfortable □Acceptable □a bit uncomfortable □ Uncomfortable

1. **What do you think of the current outdoor environment：**
2. **Temperature：**

□Very cold □quite large □ cold □moderate □slightly hot □quite hot □very hot

1. **Humidity：**

□Very dry □quite dry □slightly dry □moderate □slightly humid □quite humid □very humid

1. **Wind speed：**

□very high □quite high □slightly high □moderate □slightly small □quite small □very small

1. **Sunshine：**

□Over exposure □moderate □lack

1. **What are your expectations for thermal comfort in the current outdoor environment：**

**Temperature**： □much higher □some remain □unchanged □some lower □much lower

**Humidity**： □much larger □some larger □unchanged □some smaller □much smaller

**Wind speed**： □much larger □some larger □unchanged □some smaller □much smaller

1. **What do you think is the main factor affecting outdoor thermal comfort ：**
2. Temperature B. humidity C. wind environment D. direct sunlight E. activity intensity

F. clothing condition

**Sort：**

**【Recorder fills in】**

(1) Test area: Village testing point

(2) Test time:

(3) Test weather: □sunny □cloudy □snowy □rainy

(4) Outdoor air temperature (℃):

(5) Outdoor air humidity (%):

(6) Outdoor air velocity (m/s):
