# Supplementary figures and images for: A Study on the spatial form of traditional villages in jiangnan region of china from the perspective of human thermal comfort: A case study of nanjing, jiangsu province
Source: PLoS One. 2025 May 9;20(5):e0323252. doi: 10.1371/journal.pone.0323252 (PMC12064036; doi:10.1371/journal.pone.0323252)

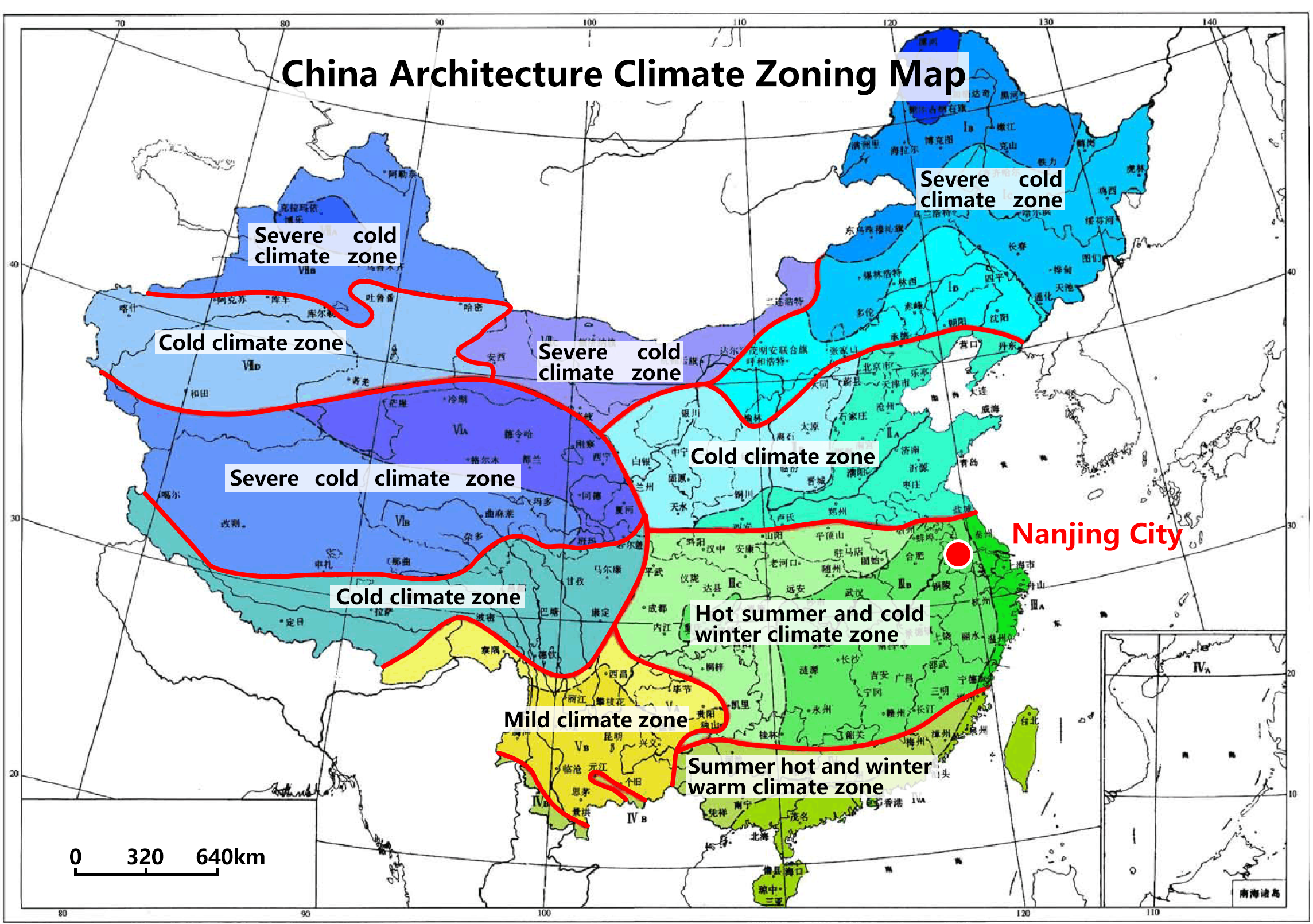

Supplement: Supporting information — (ZIP) [file pone.0323252.s001.zip › Supporting Information/S1_fig.tif]

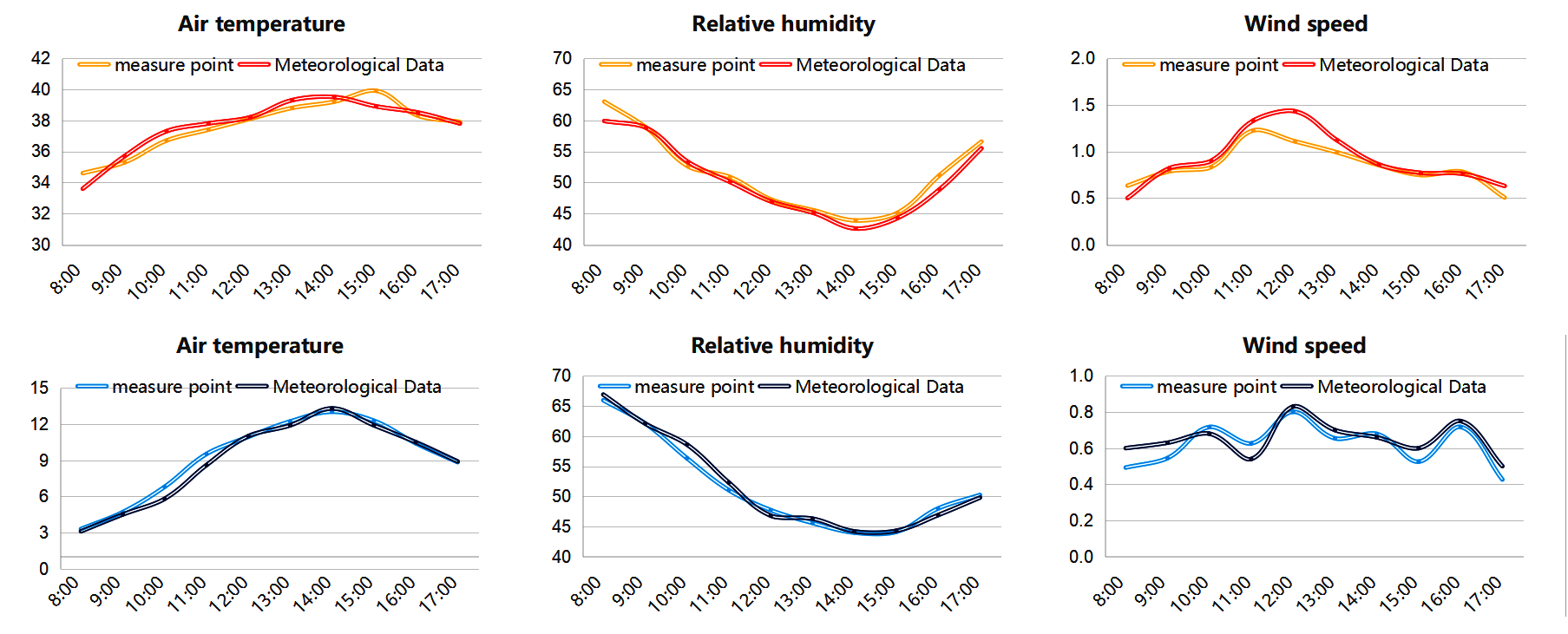

Supplement: Supporting information — (ZIP) [file pone.0323252.s001.zip › Supporting Information/S5_fig.tif]
